# Biogenic Amine Sensing Based on Rosamine a *N*-Methylpyridinium Derivative Supported on Silica Materials from Rice Husk

**DOI:** 10.3390/s22249573

**Published:** 2022-12-07

**Authors:** Carla Queirós, Susana O. Ribeiro, Ana M. G. Silva, Andreia Leite

**Affiliations:** LAQV-REQUIMTE, Departamento de Química e Bioquímica, Faculdade de Ciências, Universidade do Porto, 4169-007 Porto, Portugal

**Keywords:** biogenic amines, rice husk, silica materials, fluorescent composites, sensing, reversibility

## Abstract

In this work new rosamine–silica composites were prepared and their sensing ability towards different amines was assessed. Rice husk wastes were used as a biogenic silica source. Silica was extracted by thermal treatment, before rice husk ash and after acid leaching with citric acid–treated rice husk ash. Mesoporous material (SBA-15) was also prepared using the extracted silica. The prepared materials were characterized by several techniques such as FTIR, XRD, SEM and N_2_ adsorption. The materials were then used as adsorbents of the chromophore *N-*methylpyridinium rosamine (Ros4PyMe). The obtained loaded composites were tested in solution for amines sensing (*n-*butylamine, aniline, putrescine and cadaverine). The detection studies were analyzed by fluorescence and revealed 40% and 48% quenching in fluorescence intensity for the composite Ros4PyMe@SBA in the presence of the biogenic amines cadaverine and putrescine, respectively. The composite was also sensitive in the powder form, changing the color from violet to pale pink in the presence of putrescine vapors with a fast response (around 2 min), the process being reversible by exposure to air.

## 1. Introduction

Biogenic amines (BAs) are low-molecular-weight compounds produced by specific microorganisms (bacteria, yeasts and molds) and can be formed during storage and processing of food. High levels of BAs indicate spoilage of the food and result in bad-tasting food and foul-smelling odor [1]. The ingestion or inhalation of BAs can cause food poisoning and gastrointestinal problems among other risks for human health so it’s essential to develop appropriate probes to detect the presence of low quantities of these compounds and guarantee food safety [2]. Although BAs can be detected by several analytical methods such as: gas chromatography-mass spectrometry (GC-MS) [3], spectrophotometry [4,5], and high-performance liquid chromatography (HPLC) [6,7,8], these methods are limited by exigent sample preparation, time-consuming procedures and expensive instrumentation.

As an alternative to the previously mentioned methods are chromophore-based probes [9]. These probes have emerged as a rapid, simple and accurate molecular tool to detect trace amounts of BAs either in solution or in gaseous phase [10,11,12]. The detection is based in the alteration of their photophysical properties, namely color and fluorescence, and are particularly attractive due to their high sensitivity, direct and fast real-time monitoring using simple and low time-consuming techniques. The immobilization of these probes in solid supports can avoid precipitation problems, maintaining their fluorescence properties while allowing their in situ application and reusability.

Silica is one of the most used solid supports due to its unique features. This material can be found in many agricultural wastes namely rice husks, which are one of the richer sources of silica [13,14]. The use of extracted silica from agro-wastes provides an environmental friendly and cheap source of silica reducing production cost of silica materials and minimizing the negative environmental impact of agro-wastes disposal [15].

In this work, biogenic silica was extracted from rice husk wastes and used both as solid material and silicon source in the preparation of a mesoporous silica. To provide the optical properties to the silica material, a rosamine chromophore-based probe was used. Rosamine is a versatile fluorophore with several advantages, including high fluorescence quantum yield, good photostability and can be easily synthetized. Additionally, this positively charged chromophore possesses absorption and fluorescence spectra in the visible region, which is beneficial for visual detection.

**Figure 1:**
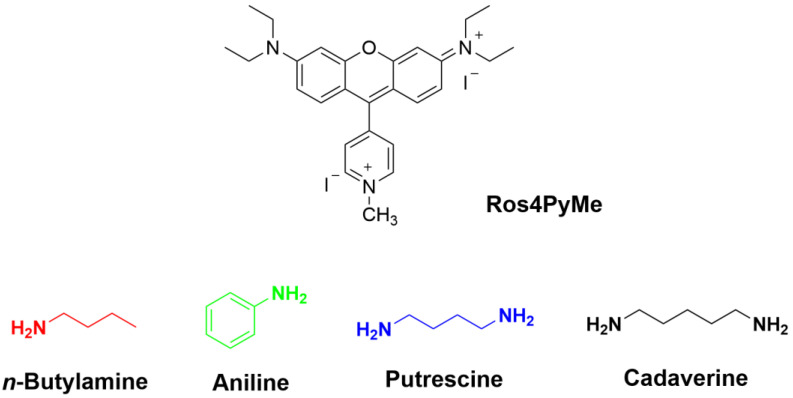
Chemical structures of *N*-methylpyridinium rosamine (Ros4PyMe, **top**) and the selected amines for the sensing studies (**bottom**)—*n*-butylamine, aniline, putrescine and cadaverine.

In a previous work our group published the synthesis and solution studies of a series of pyridyl analogs of rosamines [16]. In this work a highly reactive *N*-methylpyridinium rosamine, Ros4PyMe (Figure 1, top) showed an extinction of color and fluorescence in ethanol. This phenomenon resulted from the nucleophilic addition of the ethoxide ion to the central 9-position of the xanthene and the phenomenon was reversible by adding a weak acid. Taking in consideration that this mechanism could also occur with other nucleophilic species, such as amines, in this study the dicationic Ros4PyMe chromophore was supported in three silica materials and the sensing behavior of the resulting composites was evaluated towards various amines, namely *n-*butylamine, aniline, putrescine and cadaverine (Figure 1).

## 2. Materials and Methods

Solvents and reagents were purchased as reagent-grade from commercial suppliers and used without further purification unless otherwise stated.

Rice husk was supplied by Novarroz—Produtos Alimentares, S.A. (Oliveira de Azemeis, Portugal), and the post-treatment was performed as described in the Experimental section.

FTIR spectra were obtained on a Jasco 460 Plus spectrometer using KBr pellets, in the range 4000–400 cm^−^^1^, 64 scans.

A high-resolution (Schottky) scanning electron microscope with X-ray microanalysis and electron backscattered diffraction analysis Quanta 400 FEG ESEM/EDAX Genesis X4 M were used for SEM/EDS (Scanning electron microscopy/Energy dispersive X-ray spectroscopy studies at Centro de Materiais da Universidade do Porto. Conditions of sputtering in a SPI Module Sputter Coater equipment: powder samples coated with an Au/Pd thin film.

A Rigaku Smartlab diffractometer was used for Powder X-ray diffraction (XRD) patterns. Conditions: Bragg-Brentano para-focusing geometry using room temperature; D/tex Ultra 250 detector; Cu K-α radiation (K*α*1 wavelength 1.54059 Å); 45 kV, 200 mA; range 0.3 ≤ 2*θ* ≤ 10° (continuous mode).

For BET surface area (SBET) calculus: relative pressure data in the 0.05–0.3 range. The evaluation of total pore volume (*V*_p_) was based on the amount adsorbed at a relative pressure of about 0.95. The BJH method with the modified Kelvin equation and a correction for the statistical film thickness of the pore walls was employed for pore size distributions (obtained from the adsorption branches of the isotherms). The Harkins-Jura equation was used to calculate the film thickness (p/p_0_ range of 0.3–1.0).

Microwave-assisted rice husk chemical leaching was carried out in a MARS 6 microwave from CEM Corporation in the open vessel mode using a 3000 mL round-bottomed flask.

Electronic absorption spectra were recorded on a Shimadzu–UV 3600 UV–Vis-NIR equipped with a Shimadzu TCC-Controller (Canby, OR, USA), at 25 °C, in 1 cm cuvettes, in the wavelength range 225–700 nm. For the spectroscopic properties of Ros4PyMe a stock solution was prepared in dimethylsulfoxide (DMSO) and diluted in water, with the final concentration of DMSO below 1% (concentration range from 10^−5^–10^−7^ moldm^−3^ for the determination of the molar-absorptivity coefficient (*ε*)).

Fluorescence measurements were performed in a HORIBA Fluorolog-QM (Kyoto, Japan) equipped with a constant temperature cell holder, at 25 °C, in 1 cm cuvettes. All photophysical assays were performed under controlled temperature conditions (25 °C), using the maximum *λ*_abs_ and the appropriate *λ* range, for each Ros4PyMe@adsorbent.

Time-resolved fluorescence measurements were performed on a FluoroHub—TemPro single photon counting controller—from Horiba Jobin Yvon (Kyoto, Japan) attached to a temperature controller from JULEBO. The fluorescence excitation was performed with a NanoLED source of 562 nm from Horiba and fluorescence emission was recorded at the maximum *λ* for each solution. Time resolved experiments were recorded using a window of 4024 channels with a time calibration of *τ* 0.005 ns/channel and a peak count of 10 k. The lamp profile was recorded by inserting a scatter (diluted suspension of Ludox in waster) in place of the sample, and those results were taken into account when performing the data analysis using a nonlinear least squares iterative convolution method with the DAS6 v6.5 decay analysis software provided by Horiba Jobin-Yvon (Japan).

Fluorescence quantum yields were determined using a Quantaurus-QY C11347-11 spectrometer, absolute luminescence quantum yield spectrometer Hamamatsu Photonics (Massy, France).

### 2.1. Synthesis of Ros4PyMe

The synthesis of *N*-methylpyridinium rosamine—Ros4PyMe—was based on a previous report [16].

### 2.2. Silica Materials Preparation

Preparation of the silica materials involved several steps. Primarily, the rice husk (RH) was washed with water to remove soil and dust and dried at 100 °C for 2 h. To obtain rice husk ash (RHA), under controlled conditions, the RH was calcinated at 600 °C for 4 h (ramp of 5 °C min*^−^*^1^). Treated rice husk ash (TRHA) was obtained by performing a chemical leaching using a 5% citric acid solution under microwave irradiation (MW) 100 W at 95 °C during 30 min, followed by heat treatment at 600 °C for 4 h (ramp of 5 °C min*^−^*^1^). 

Sodium silicate solution was prepared by refluxing under MW irradiation RHA in 1 moldm*^−^*^3^ NaOH in H_2_O at 100 °C, 100 W for 15 min, followed by filtration.

Santa Barbara Amorphous-15 (SBA-15) was synthesized according to the procedure described by Bhagiyalakshmi et al. [17], using a triblock copolymer, Pluronic P123. Pluronic P123 (1.0 g) was dissolved in aqueous HCl (1.6 moldm*^−^*^3^, 38 mL) under stirring at 40 °C and 40 mL of the sodium silicate solution was added dropwise. The mixture was stirred for 24 h at 40 °C in a Teflon autoclave and then raised to 100 °C for another 24 h. The resulting product was filtered, dried, and the organic template was removed through calcination at 550 °C for 5 h with a ramp of 1 °C min*^−^*^1^.

### 2.3. Composites Preparation

Several assays were conducted to determine the adsorption capacity, *q*, of the different adsorbents for Ros4PyMe in the concentration range previously reported [16]. In these assays’ addictive amounts of a stock solution of Ros4PyMe in water were added to a dispersion of 50 mg of adsorbent in 2 mL of water. Between each addition the dispersion was stirred for 2 min, centrifuged for 1 min at 13,400 r.p.m. and the supernatant color observed. When a violet coloration was detected in the supernatant, the additions were stopped, and the composites were washed twice with water and dried overnight under vacuum (50 mbar, 75 °C). The concentration of adsorbed Ros4PyMe was calculated using the calibration curve of Ros4PyMe in water obtained by UV–Vis spectroscopy.

### 2.4. Biogenic Amines Detection

For the amine detection assays 1.4 mg of the Ros4PyMe@adsorbent composites were dispersed by ultrasounds (10 min) in 5 mL of water. Interaction of the dispersion with the studied amines was evaluated using fluorescence measurements in water at 25 °C. Stock solutions of the different amines—*n*-butylamine, aniline, cadaverine, and putrescine—prepared in water were added to the dispersion up to an amine concentration of 10*^−^*^4^ moldm*^−^*^3^. Fluorescence intensities were always corrected for dilution. Leaching of Ros4PyMe from the composites was verified by UV–Vis and fluorescence spectroscopies at the end of each assay, after centrifugation of the dispersion. For control purposes the interaction behavior of Ros4PyMe with putrescine was also evaluated, using a Ros4PyMe concentration of 2.5 × 10*^−^*^6^ moldm*^−^*^3^.

## 3. Results

### 3.1. Ros4PyMe Spectroscopic Properties

To assess the optical spectroscopic properties of rosamine Ros4PyMe, in the same experimental condition that would be used in the amine detection studies, UV–Vis absorption and fluorescence emission measurements were performed in water as solvent. The compound presents: *λ*_abs max_ = 580 nm, *λ*_em_ = 672 nm (Figure S1), *ε* = 6.540 × 10^4^ mol*^−^*^1^ dm^3^ cm*^−^*^1^, and *Φ_F_* = 0.028.

The calibration curve for Ros4PyMe, in water and within a 5.0 × 10*^−^*^7^ to 1.6 × 10*^−^*^5^ moldm*^−^*^3^ concentration range, was obtained and its linear regression represented by the equation:

A = 6.540 × 10^4^ |Ros4PyMe| − 0.0209 (R^2^ = 0.998)

The equation was used in the determination of the quantity of Ros4PyMe adsorbed by the silica materials.

### 3.2. Ros4PyMe Composites

Preliminary studies indicate that a contact time between Ros4PyMe and the adsorbents of 2 min, under stirring at 600 r.p.m., was sufficient for the adsorption of small amounts of Ros4PyMe. The amount of Ros4PyMe used was chosen taking in consideration the data previously reported, to avoid fluorescence quenching due to inner filter effect. Considering the results these conditions were used in the adsorption assays.

Using the Ros4PyMe calibration curve and the UV–Vis spectra of the solution after the adsorption studies for each adsorbent, the adsorption coefficient (*q*), the concentration of Ros4PyMe adsorbed and the corresponding adsorbed % were calculated. The adsorption capacity (*q*) [18], in mg/g, for each adsorbent was determined using the Equation (1):

*q* = [(*C*_0_ − *C_e_*)/*m*] × *V* (1)

where *C*_0_ and *C_e_* are the initial and equilibrium concentration of Ros4PyMe in mg/L, respectively, *m* is the adsorbent mass in g, and *V* represents the dispersion volume in L.

The adsorption results are presented in Table 1. Analysis of the results clearly show that the silica-based material SBA-15 is the best adsorbent for Ros4PyMe, followed by RHA and TRHA. With SBA-15, both the adsorption coefficient and the adsorbed concentration of Ros4PyMe is around 2.4 and 8.1 times higher than the corresponding values for RHA and TRHA, respectively.

Regarding the adsorbents RHA and TRHA other factors are influencing the adsorption of the rosamine, since that for TRHA the values of specific surface area and total volume are higher than for RHA, but smaller adsorption values are obtained. In a previous reported study by Santana et al. [19], using rice husks as adsorbents, the authors reported that the pH at point of zero charge of the adsorbent decrease from 5.3 in natura rice husk to 3.8 after acid treatment, indicating a change in the surface characteristics with an increased number of protonated sites. In this work, the acidic treatment of the rice husks in the preparation of the TRHA adsorbent seems to have the same effect, leading to the protonation of the reactive sites, decreasing the ability of the material to adsorb the dicationic Ros4PyMe.

All composites Ros4PyMe@adsorbent present a violet coloration and different fluorescence intensity under UV light (Figure S2 in the Supplementary Materials). The composites UV–Vis absorption and fluorescence maximum wavelengths, *Φ_F_*, and fluorescence lifetime (*τ*) are presented in Table 2.

UV–Vis absorption and fluorescence maximum wavelengths present small shifts comparing to those of Ros4PyMe. These shifts are more accentuated in the absorption band, with redshifts of 15 nm, 24 nm and 12 nm for RHA, TRHA and SBA composites, respectively, and indicates the existence of some degree of interaction between the rosamine derivative and the adsorbents. The shift in the emission band is less accentuated, which can be explained by the fact that the emission comes only from Ros4PyMe, no contribution is associated to the adsorbent. The fluorescence quantum yields of the composites are higher than that of Ros4PyMe revealing a higher structural rigidity of the chromophore when adsorbed in the silica materials. This is confirmed by the fluorescence lifetime values, which are slightly higher for the composites.

The presented results confirm the adsorption of the Ros4PyMe by all the silica materials and show that there are significant improvements in the composites spectroscopic properties in comparation with the not immobilized Ros4PyMe, as can be confirmed by the increase in the fluorescence quantum yield and lifetime values.

### 3.3. Ros4PyMe@SBA Characterization

The adsorption results can be related with the characteristics of each silica material, so the prepared materials were characterized by nitrogen adsorption/desorption isotherms, XRD, FTIR, and SEM analyses. Considering the results obtained in the previous studies, only the composite Ros4PyMe@SBA was fully characterized, as well as the silica materials.

**Figure 2:**
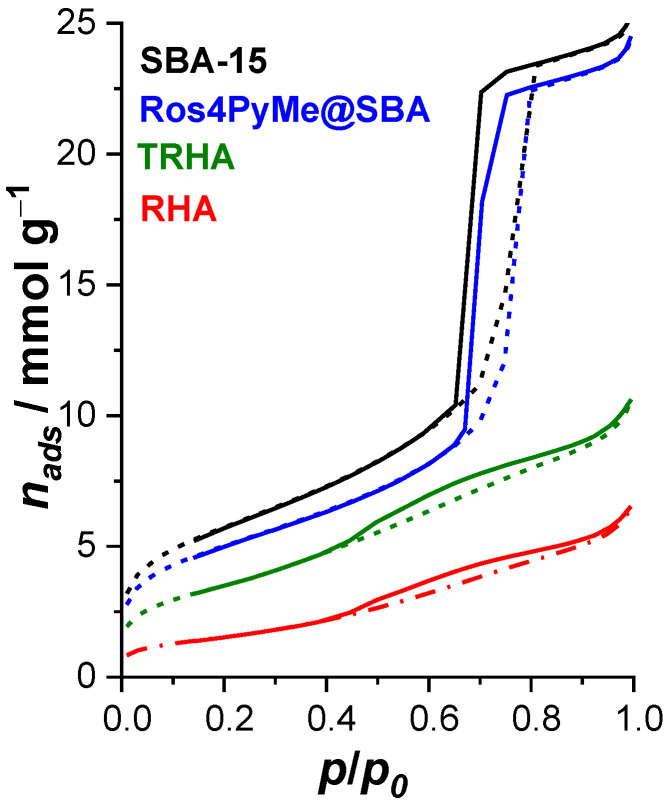
N_2_ adsorptiondesorption isotherms of RHA, TRHA, SBA-15, and Ros4PyMe@SBA (adsorption—full lines and desorption—dashed lines).

Nitrogen adsorption/desorption isotherms were obtained for the synthesized materials and are displayed in Figure 2. The RHA and TRHA adsorbents exhibit Type II isotherm with closed hysteresis loop about *p*/*p*_0_ 0.45 to 1.00, indicating the presence of less but larger meso- and macropores in the materials particles [20,21]. For the SBA-15 adsorbent, Type IV isotherms are observed, with parallel curves typical of H1 hysteresis that indicates the uniform distribution of mesopores. It can also be observed in Figure 2 that the Ros4PyMe@SBA adsorbed retain the Type IV isotherm shape of the bare SBA-15. The specific surface area and total volume of the materials increase in the order RHA, TRHA, and SBA-15 (Table 3), and a slight decrease is observed for the composite Ros4PyMe@SBA, due to the adsorption of Ros4PyMe in the SBA-15 pores.

**Figure 3:**
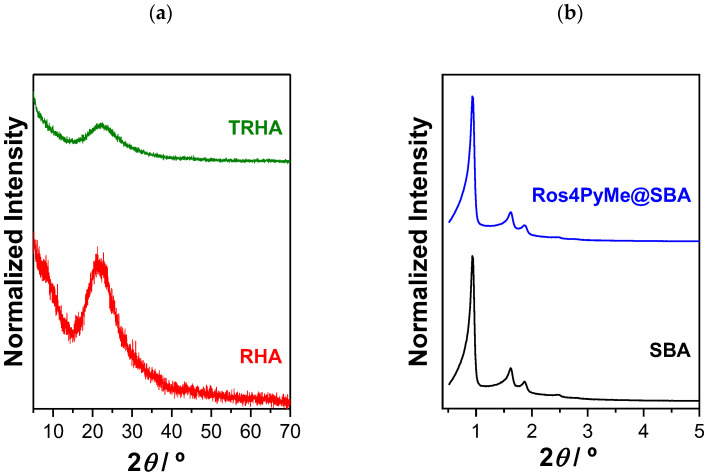
XRD patterns of RHA and TRHA (**a**) and SBA-15 and Ros4PyMe@SBA (**b**).

XRD patterns of the different adsorbents and Ros4PyMe@SBA composite are depicted in Figure 3. Analysis of the XRD patterns of RHA and TRHA indicate that these materials are amorphous in nature, and no evidence of the presence of crystalline phases was detected from the XRD patterns, in accordance with previous publications that refer the amorphous nature of silica particles derived from rice husks [22,23]. The XRD pattern of SBA-15 presents three well-resolved peaks corresponding to the (100), (110), and (200) reflections of a *p6mm* hexagonal symmetry characteristic of this mesoporous material, which confirms its successful preparation [17,24]. The similarity of the SBA-15 XRD patterns before and after Ros4PyMe adsorption indicates that the highly ordered SBA-15 mesostructure was preserved upon adsorption of the rosamine.

**Figure 4:**
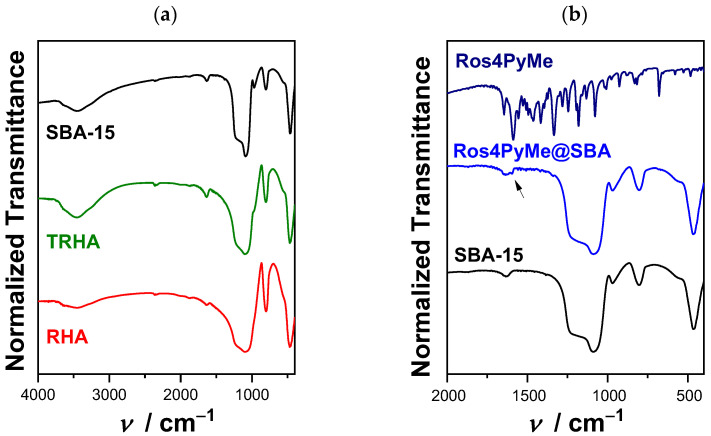
FTIR spectra of: (**a**) RHA, TRHA, and SBA-15 in the range 4000–400 cm^−1^ and (**b**) SBA-15, Ros4PyMe@SBA, and Ros4PyMe between 2000 and 400 cm^−1^.

FTIR spectra of the prepared materials presented in Figure 4 reveal the characteristic bands of the silica framework. These bands can be ascribed to *ν_as_* (Si-O-Si), *ν_s_* (Si-O-Si), and *δ*(O–Si–O) located around 1083, 802, and 458 cm^−1^, respectively [19]. Additionally, characteristic bands of the vibration and stretching modes of the (OH) bond, with frequencies in the regions of 1625 cm^−1^ and 3100–3600 cm^−1^ due to the presence of adsorbed water in the samples and silanol groups (Si-OH) are also observed. The silanol groups (Si-OH), with a frequency at 970 cm^−1^, are more evident in the SBA-15 material. No significant differences are observed between the SBA-15 and Ros4PyMe@SBA FTIR spectrum, except for a broadening of the band around 1596 cm^−1^, which can be attributed to the presence of Ros4PyMe in the composite [20,25].

**Figure 5:**
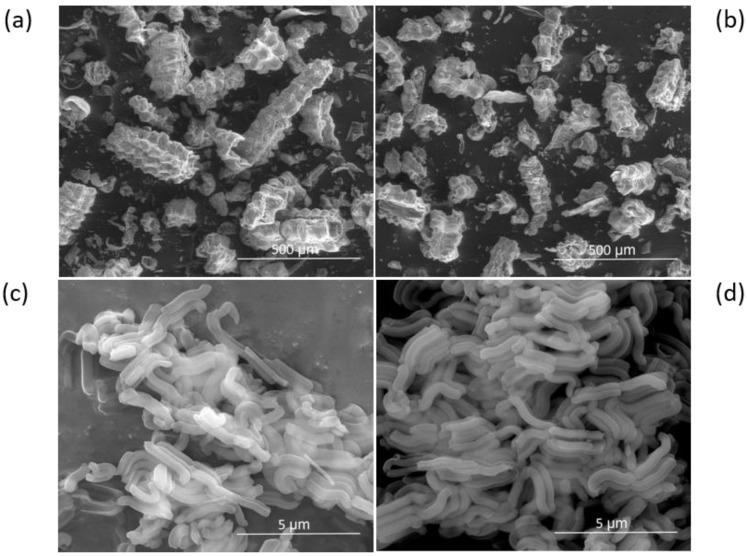
SEM images of RHA (**a**), TRHA (**b**), SBA-15 (**c**), and Ros4PyMe@SBA (**d**).

The morphology of the prepared materials was observed by SEM analysis and is presented in Figure 5. RHA and TRHA particles present the typical structure of rice husks skeleton. It can also be observed that TRHA present particles with smaller diameter than RHA, which can be attributed to the reduction of the mean particle diameter during acid leaching. Through the microstructural analysis, it was possible to observe the long-rod morphology with relatively uniform sizes for SBA-15, which was maintained after the Ros4PyMe adsorption.

### 3.4. Amines Sensing

According to several studies [26,27,28], mesoporous silica materials are suitable adsorbents for amines and allow their detection; however, the applied sensing techniques are limited by the previous mentioned reasons [26]. Four amines were selected for the sensing studies: *n*-butylamine, aniline, and two biogenic amines, putrescine and cadaverine (Figure 1). *N*-butylamine and aniline were used to access if there was a preference for aliphatic and aromatic amine and if the higher steric hindrance and lower nucleophilicity of aniline would affect the sensitivity. The biogenic amines were selected due to the high interest in the control of their concentrations in food products and in how those products deteriorate along time (shelf time).

#### 3.4.1. Amines Sensing Studies

**Figure 6:**
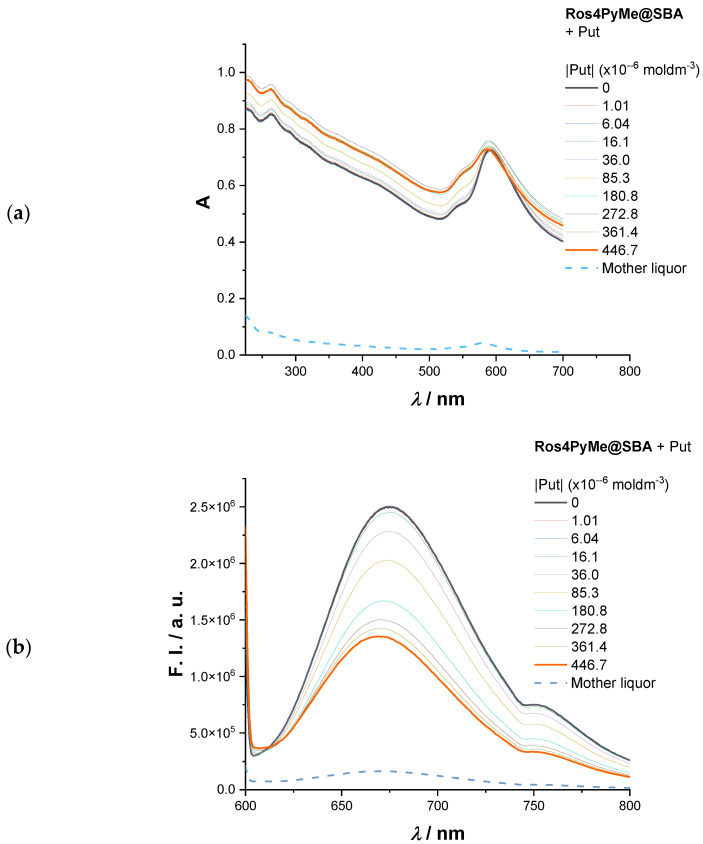
Absorption (**a**) and emission (**b**) spectra of Ros4PyMe@SBA with increasing amounts of putrescine (up to 4.47 × 10^−4^ moldm^−3^) in water at 25 °C.

Sensing studies were performed by the addition of increasing amounts of amine to a dispersion of Ros4PyMe@adsorbent composite in water every 2 min, under stirring, followed by absorption and fluorescence intensity (F. I.) measurements. After the assays, the dispersions were centrifuged, and the supernatant was analyzed by UV–Vis to evaluate the potential leaching of Ros4PyMe from the composites. Figure 6 depicts the absorption and emission spectra of Ros4PyMe@SBA composite in the presence of putrescine. The obtained data is used as an example to show the effect observed after the amine additions to the absorption and emission spectra of Ros4PyMe@SBA composite.

Analysis of the absorption spectra reveals a small change in the intensity and maximum absorption wavelength regarding the band attributed to the pyridyl unit (Ros4PyMe) and negligible leaching of the fluorophore—reflected in the very low F. I. value. On the other hand, a significant quenching of the fluorescence intensity is observed with the increase of putrescine concentration, with a total quenching around 50%. Table 4 summarizes the results attained for the three composites in the presence of the studied amines. Mainly the values present a decrease (quenching) in the F. I. intensity comparing the initial value (absence of amine) and final value (presence of 4.43 × 10^−4^ moldm^−3^ of the amine). Aniline was only used in the case of Ros4PyMe@SBA, considering this seemed the most promising material.

In a previous work, but using other rosamine analogue, the same preference for the studied amines was observed: *n*-butylamine > cadaverine > putrescine [29]. The sensitivity of Ros4PyMe@SBA to the tested amines can be related not only with their nucleophilicity, that follows the order: aniline < cadaverine < *n*-butylamine = putrescine [30], but also with the preference for the presence of two amino groups in the amine structure which explains the fact that the sensor responds better to putrescine than *n*-butylamine.

**Figure 7:**
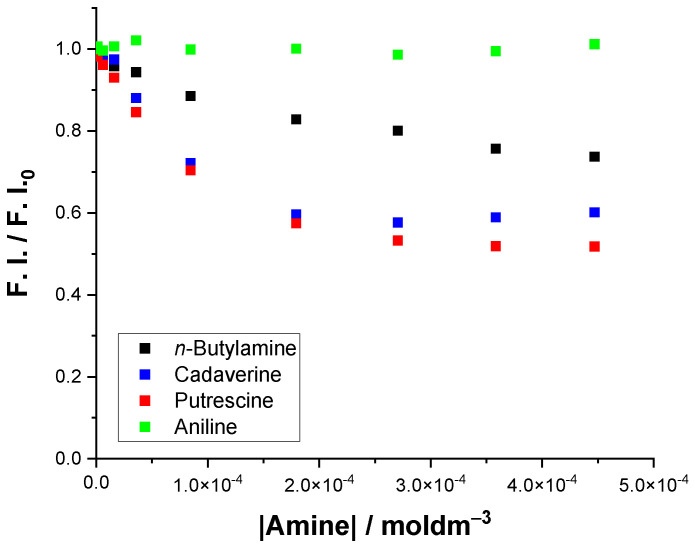
Fluorescence intensity variation of Ros4PyMe@SBA in the absence (F. I._0_), and presence of increasing amounts (up to 4.47 × 10^−4^ moldm^−3^) of the selected amines (*n*-butylamine, cadaverine, putrescine and aniline) in water at 25 °C.

Figure 7 shows the variation in the fluorescence intensity (F. I./F. I._0_) for Ros4PyMe@SBA in the presence of the study amines. Data shows a decrease of the F. I. with the increase in amine concentration with a stabilization for concentration around 2 × 10^−4^–3 × 10^−4^ moldm^−3^ (values below 50 ppm for the amines), with the exception of aniline. In the case of aniline, the variation in the F. I. was neglectable (around 1%).

The data analysis reveals that Ros4PyMe@SBA is, in general, the most sensitive composite towards the studied amines, namely the biogenic amines, confirming that the silica material and consequently the corresponding composite properties are crucial for a better sensitivity towards the analyte’s detection. Although an increase in the fluorescence intensity is observed for Ros4PyMe@TRHA, the values are less significative that those determined for Ros4PyMe@SBA. The results attained for Ros4PyMe@SBA in the sensing of putrescine and cadaverine are very promising and bellow the maximum tolerable limits for sauerkraut, fish, cheese, fermented sausages and seasonings (all above 100 ppm) [31]. For the maximum concentrations tested for putrescine and cadaverine the ppm values are of 43.3 and 49.8, respectively. These low limits can be advantageous to study, variations/increases in the amine quantities during the shelf life of food products, like atmosphere packed pork cutlets [32].

A control assay, similar to the previous one, was performed to access the sensitivity of the chromophore Ros4PyMe towards putrescine (Figure S3 in Supplementary Materials). The collected data regarding the spectroscopic properties of the chromophore reveal only small variations in the intensity values (absorption and emission) after the putrescine addition, which lead to the conclusion that the composite presents higher sensitivity towards the amine.

The sensitivity of Ros4PyMe@SBA and Ros4PyMe towards putrescine, defined by the limits of detection (*LoD*) and quantification (*LoQ*), was determined through the calibration curve method for the fluorescence spectral responses. Equations (2) and (3) were employed for the values determination [33]:

(2) LoD=3.3×σ×Ns

(3) LoQ=10×σ×Ns

where *σ* (standard error) represents the standard deviation of the response, *s* is the slope of the calibration curve, N is the number of independent repetitions (*N* = 3) and the coefficients 3.3 and 10 are expansion factors obtained assuming a 95% confidence level [34]. The *LoD* (and *LoQ*) values of putrescine detection for Ros4PyMe@SBA were calculated to be 7.86 μmol dm^−3^ and (23.8 μmol dm^−3^). No *LoD* could be determined for Ros4PyMe considering that its fluorescence response with increasing amounts of putrescine is not linear. These results show once more that the adsorption of Ros4PyMe in the silica matrix induces an improvement in the sensing properties allowing its use in the detection of biogenic amines.

#### 3.4.2. Ros4PyMe@SBA-Sensing Behavior towards Putrescine Vapor

**Figure 8:**
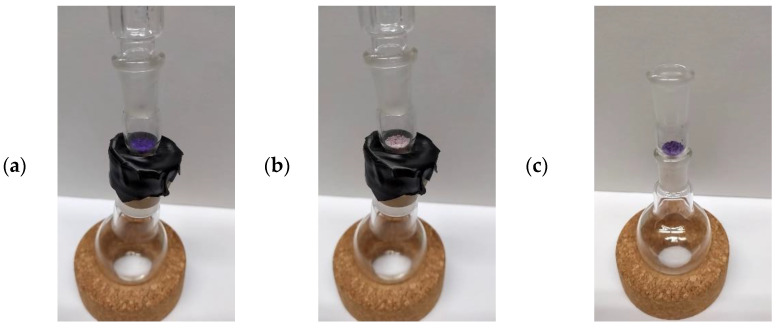
Photographs of Ros4PyMe@SBA after exposure to putrescine (vapor) at time zero (**a**), after 2 min (**b**) and after exposure to air (approximately 2 min)—removal of the amine and open system (**c**).

Chemosensors that allow to perform sensing studies both in solution and in solid supports are of great interest since that increases their applicability. With this in mind we decided to test the composite Ros4PyMe@SBA powder sensitivity towards putrescine vapors. A simple apparatus (Figure 8) was used, in which 2 mL of putrescine were placed in the round bottom balloon and Ros4PyMe@SBA was placed in the top on a glass adapter. The system was closed and in less than 2 min a significant change in the powder color—from violet to pale—was observed (Video S1 in the Supplementary Materials). This result showed that the composite in the powder form is highly sensitive to putrescine vapors, presenting a fast response in its presence. The next step was to evaluate the composite recovery after the exposure, the system was open, the round bottom balloon replaced by an empty one and the regain of the violet color of the powder was observed within a 2.5 min time frame to air exposure (Figure 8; Video S2 in Supplementary Materials). Other cycles of exposure to putrescine/exposure to air were tested (up to three times) and the same behavior was observed. Further studies are needed to determine the minimum amine vapor concentration to observe the color change.

### 3.5. Mechanism Hypothesis

According to our previous studies [16] the previously synthesized chromophore Ros4PyMe presents strong susceptibility to react with nucleophilic species, through a nucleophilic addition reaction to the central 9-position of the xanthene, and this reactivity seems to be maintained or even improved in the Ros4PyMe@SBA composite. The loss of color is due to the interruption of the *π*-conjugation of the xanthene moiety, being verified that the process is reversible by the evaporation of the amine vapors, after exposure to air.

## 4. Conclusions

This work describes the preparation of new rosamine–silica composites and their sensing ability towards the biogenic amines cadaverine and putrescine.

The rice husk wastes derived materials RHA and TRHA and the mesoporous silica SBA-15 were used as adsorbents for *N-*methylpyridinium rosamine (Ros4PyMe), originating the Ros4PyMe@adsorbent composites. SBA-15 proved to be the best adsorbent for Ros4PyMe with an adsorption capacity around 2.4 and 8.1 times higher than the corresponding values obtained for RHA and TRHA, respectively.

The detection amines studies with composite Ros4PyMe@SBA revealed a quenching in fluorescence intensity of 40% for cadaverine and 48% for putrescine. The maximum concentrations tested for putrescine and cadaverine, 43.3 ppm and 49.8 ppm, respectively, are below the maximum tolerable limits for some common food products, such as fish, cheese, which enable the application of this composite to monitor the presence of these amines during the shelf life of these products. The composite was also sensitive in the powder form, changing the color from violet to pale pink in the presence of putrescine vapors with a fast response (around 2 min), being the process reversible.

From the results obtained, we can conclude that the immobilization of the Ros4PyMe on mesoporous silica SBA-15, allows to obtain a new fluorescent material with high sensitivity to biogenic amines, allowing its application as a sensor both in solution and in solid state.

## Figures and Tables

**Table 1:** Adsorption coefficient, adsorbed concentration of Ros4PyMe and % of adsorbed Ros4PyMe.

Silica-Based Material	*q* (mg/g)	|Ros4PyMe|_adsorbed_ (×10^−3^ moldm^−3^)	Adsorbed Ros4PyMe (%)
RHA	12.3	0.478	99.7
TRHA	3.72	0.138	98.9
SBA-15	30.1	1.120	99.7

**Table 2:** Spectroscopic properties of Ros4PyMe and Ros4PyMe@adsorbent composites in water and 25 °C.

Composite	*λ*_abs max_ (nm)	*λ*_em_ (nm)	*Φ_F_* (%)	*τ* (ns)
Ros4PyMe	580	673	2.0 [16]	0.070 ± 0.009
Ros4PyMe@RHA	595	674	3.9	0.10 ± 0.01
Ros4PyMe@TRHA	604	678	7.8	0.10 ± 0.02
Ros4PyMe@SBA	592	675	5.1	0.12 ± 0.02

**Table 3:** Textural parameters of RHA, TRHA, SBA-15, and Ros4PyMe@SBA.

	*S*_BET_ (m^2^ g^−1^)	V_total_ (cm^3^ g^−1^)
RHA	118.91	0.19
TRHA	272.78	0.32
SBA-15	446.09	0.84
Ros4PyMe@SBA	391.01	0.81

**Table 4:** F. I. variation of the composites—Ros4PyMe@RHA, Ros4PyMe@TRHA, and Ros4PyMe@SBA — in the presence of the selected amines (|Amine|_max_ = 4.47 × 10^−4^ moldm^−3^) in water and 25 °C.

	F. I. Variation/%		
Amine	Ros4PyMe@RHA	Ros4PyMe@TRHA	Ros4PyMe@SBA
*n*-Butylamine	30	1 *	26
Putrescine	0.7	19 *	48
Cadaverine	0.2	9	40
Aniline	-	-	1

* These values represent an increase in the F. I. values—enhancement.

## Data Availability

Not applicable.

## Supplementary Materials

The following supporting information can be downloaded at: https://www.mdpi.com/article/10.3390/s22249573/s1: Figure S1: Normalized absorption (black line) and emission (red line) spectra of Ros4PyMe in water at 25 °C (normalization was done at the maximum wavelengths, *λ*_abs_ = 580 nm and *λ*_em_ = 673 nm). Figure S2: Photographs of the prepared silica materials (RHA, TRHA, and SBA-15, top powders) and powder composites (Ros4PyMe@RHA, Ros4PyMe@TRH, and Ros4PyMe@SBA, bottom powders) at the naked eye (left) and under UV light (right). Figure S3: Absorption (top) and emission (bottom) spectra of Ros4PyMe in the presence of increasing amounts of putrescine in water at 25 °C. Video S1: Putrescine sensing. Video S2: Reversibility.
