# Supplementary material for: Biogenic Amine Sensing Based on Rosamine a N-Methylpyridinium Derivative Supported on Silica Materials from Rice Husk
Source: Sensors (Basel). 2022 Dec 7;22(24):9573. doi: 10.3390/s22249573 (PMC9784884; doi:10.3390/s22249573)
Supplement: Supplementary file 1 [file sensors-22-09573-s001.zip › Supplementary Materials.pdf]

Supplementary Materials

Biogenic amine sensing based on rosamine a N-methylpyridinium derivative supported on silica materials from rice husk

Carla Queirós, Susana O. Ribeiro, Ana M. G. Silva, Andreia Leite*

[1]*REQUIMTE, LAQV, Departamento de Química e Bioquímica, Faculdade de Ciências, Universidade do Porto, Porto, Portugal*

*Corresponding author: Andreia Leite; E-mail: acleite@fc.up.pt

Figure S1. Absorption and emission spectra of Ros4PyMe

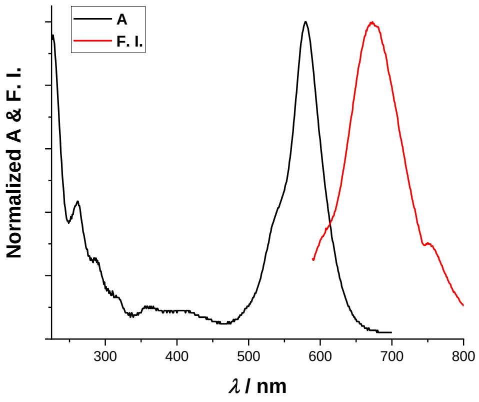

Figure S1. Normalized absorption (black line) and emission (red line) spectra of **Ros4PyMe** in water at 25 °C (normalization was done at the maximum wavelengths, λ_{abs} = 580 nm and λ_{em} = 673 nm).

Figure S2. Powder composites

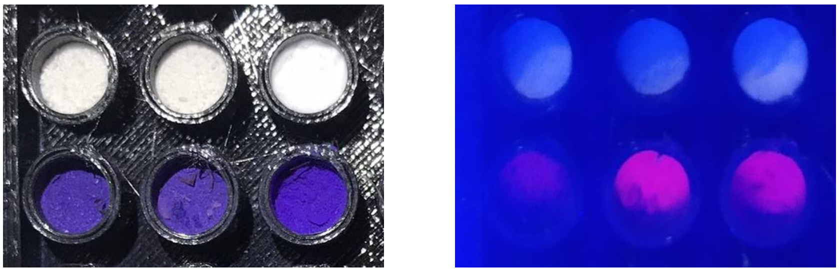

Figure S2. Photographs of the prepared silica materials (RHA, TRHA and SBA-15, top powders) and powder composites (**Ros4PyMe@RHA**, **Ros4PyMe@TRH** and **Ros4PyMe@SBA**, bottom powders) at naked eye (left) and under UV light (right).

Figure S3. Absorption and emission spectra of **Ros4PyMe** in the presence of putrescine

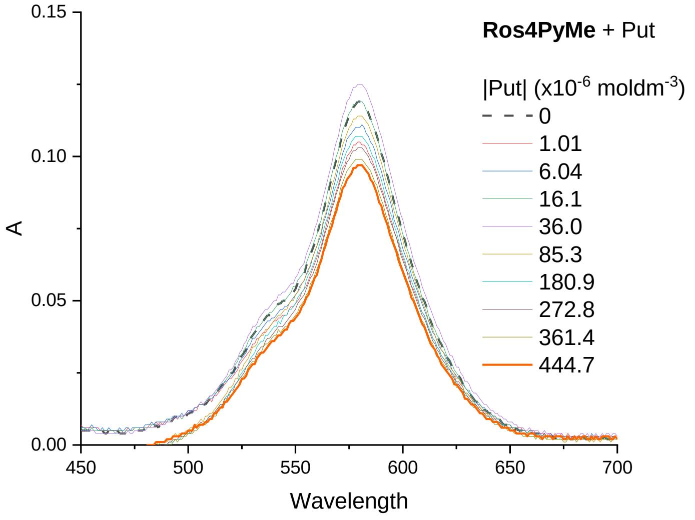

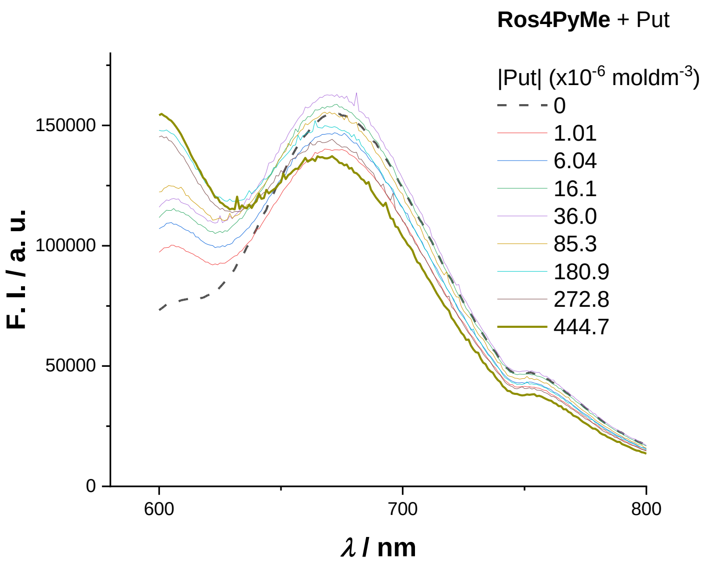

Figure S3. Absorption (top) and emission (bottom) spectra of **Ros4PyMe** in the presence of increasing amounts of putrescine in water at 25 °C.
